# Severe asymptomatic maternal antepartum hyponatremia leading to neonatal seizures: prevention is better than cure

**DOI:** 10.1186/s40748-015-0027-0

**Published:** 2015-11-04

**Authors:** Enrico Valerio, Margherita Fantinato, Irene Alba Beatrice Giovannini, Eugenio Baraldi, Lino Chiandetti

**Affiliations:** Department of Woman and Child Health, Medical School, University of Padua, Via Giustiniani, 3, 35128 Padova, Italy

**Keywords:** Hyponatremia, Neonatal seizures, Newborn, Intensive care, Neonatology, Obstetrics

## Abstract

**Background:**

Pre-delivery maternal electrolyte derangements may reflect themselves in the newborn, since placental homeostasis determines electrolyte equilibrium between mother and fetus.

**Case presentation:**

A term newborn, transferred to our Neonatal Intensive Care Unit 1 h after birth for an apnoea episode, presented with initially left-sided, and subsequently generalized tonic-clonic seizures due to severe hyponatremia (119 mmol/L). Seizures rapidly ceased after electrolyte correction plus a phenobarbital bolus. Deep hyponatremia was also detected in the mother (123 mmol/L).

**Conclusions:**

As placental homeostasis determines electrolytes equilibrium between mother and fetus, obstetrics and neonatologists should be aware that any maternal dyselectrolytemia will reflect itself in the newborn; hence, it is fundamental to detect possible maternal electrolyte imbalances before delivery, in order to be prepared to timely correction of electrolyte derangements in the newborn.

## Background

Placenta represents the key organ for maternal-fetal interaction all along pregnancy. Apart from its role in delivering nutrients to and removing waste products from the fetus -as well as its endocrine functionalityplacenta also controls micronutrient, trace element, and electrolyte transfer from mother to fetus and vice versa, maintaining homeostatic equilibrium between the two circulations. Pre-delivery maternal electrolyte derangements may reflect themselves in the newborn, since placentalhomeostasis determines electrolyte equilibrium between mother and fetus.

## Case report

A term neonate was born on vaginal, non-underwater delivery at 39 + 6 weeks. Obstetric history was remarkable for gestational diabetes controlled with diet therapy. The mother wasn’t under any medications. During labor –which lasted 17 h- she was administered parenteral hydration with 5 % dextrose, with liberalized oral water intake which was not quantifiable. No oxytocin or other medications were administered. Maternal blood pressure was normal before delivery; no other signs of pre-eclampsia were noted.

Delivery was substantially uneventful, except for lightly meconium-stained amniotic fluid. Apgar index was nine at first and ten at fifth minute. Neonatologists were not called to assist at delivery.

Umbilical cord blood gas analysis performed in delivery room showed severe hyponatriemia (sodium 117.8 mmol/L). Other electrolytes were normal. No electrolyte correction was started at this point, and the baby was given to the mother to begin skin-to-skin contact.

One hour after birth, while lying on her mother’s breast, the baby presented an apnoea episode for which neonatologists were called; at evaluation the baby presented normal air entry with expiratory grunting and O2 saturation ranging from 80 to 85 % in room air. On these basis and given the anamnestic data of meconium-stained amiotic fluid, the baby was admnistered nasal continuous positive air pressure with O2 saturation rise to 98 %, and then transferred to our Neonatal Intensive Care Unit (NICU) to start intravenous antibiotic therapy.

During umbilical vein incannulation procedure, the baby presented initially left-sided, and subsequently generalized tonic-clonic seizures. A rapid blood glucose test was obtained, showing normoglycemia (96 mg/dl); a venous blood gas analysis was performed, showing deep hyponatremia (sodium 119 mEq/L) and mild hypocalcemia (ionized calcium 1.15 mmol/L), both subsequently confirmed by laboratory analysis. Rapid electrolyte correction with two sodium chloride boluses (2 mEq/kg each, in 5 min) and one calcium gluconate bolus (0.3 mEq/kg, in 10 min, under ECG monitoring) was undertaken, along with a phenobarbital bolus (20 mg/kg, in 20 min). Seizures rapidly ceased; normal reactivity and crying were present 2 h later. Blood gas analysis undertaken 1 h after seizures start showed a sodium level of 124.4 mmol/L, which was considered a safe threshold; from that moment on, slow sodium intravenous supplementation (pure sodium chloride [concentration 2 mmol/ml], diluted with distilled water for IV use) was then started and maintained for the next 24 h. Following seriated blood gas analyses showed stabilization of sodium levels around 124 mmol/L for the next 6 h; then, blood sodium showed a steady rise and reached a new stabilization around 140 mmol/L about 20 h after the reaching of the “safe threshold” of 124.4. Based on this data, hyponatremia was corrected at the approximate velocity of 0.78 mmol/L/h.

Immediately after seizures resolution, a cerebral ultrasound was performed: no sign of edema, intracranial bleeding, or other structural anomalies were evident. Overnight cerebral function monitoring (CFM) was then started, showing no sign of gross electrical abnormalities. Electroencephalogram (EEG) was performed 12 h after initial symptoms, showing no pathological features; phenobarbital was then discontinued without seizure relapse.

No fluid restriction was employed during sodium correction; the baby was fed with normal term formula.

Urinary electrolyte excretion fraction calculated at 36 h of life was normal; blood urea nitrogen and creatinine were normal.

C-reactive protein was permanently negative during admission. Birth hemoculture turned out negative.

One-month post-discharge follow up visit showed normal neurodevelopment.

An anamnestic supplement, obtained some hours after neonatal symptoms onset, revealed severe asymptomatic maternal hyponatremia (123 mmol/L at a blood sample drawn 4 h before delivery), corrected only several hours after delivery. A maternal blood sample drawn 24 h after delivery showed normal plasma sodium (140 mmol/L). Pre-discharge control also showed maternal normonatremia.

## Discussion

### Neonatal seizures: overview

Neonatal seizures always require rapid correction in order to prevent permanent cerebral damage. Neonatal period is the one at highest risk for seizures (1.8-5/1000 live births in the US); relative incidence is higher in preterm (3.9 %) compared to term babies (1.5 %) [1]. Neonatal seizures’ etiology, semiology, and electroencephalographic features are peculiarly different from those of other age groups, and can be refractory to commonly used antiepileptic drugs.

Loman et al. [2] classified neonatal seizures etiology into hypoxic-ischemic encephalopathy, ischemic infarction, intracranial hemorrhage, intracranial infections, metabolic or electrolyte disorders, congenital malformations of the central nervous system, inborn errors of metabolism, intoxications and epileptic syndromes. In the setting of emergent, clinically unmistakable neonatal seizures with no available anamnestic clues on etiology, a symptom-based, stepwise rapid diagnostic-therapeutic algorhythm is advisable. A blood gas analysis should be obtained immediately in order to assess electrolyte and glucose status of the newborn, followed by rapid correction if needed. If no alterations in blood electrolytes or glucose are found, or if rapid metabolic correction is not yet sufficient to control seizures, antiepileptic drugs should be used: current practice includes first line therapy with phenobarbital (loading dose 20–40 mg/kg) [3], followed by phenytoin and/or benzodiazepines such as lorazepam (0.05– 0.1 mg/kg) as second line therapy to treat refractory seizures [4]. Once symptoms are under control, a thorough diagnostic work-up (including cerebral ultrasonography and/or magnetic resonance imaging, electroencephalogram, microbiology tests, laboratory ammonium and lactate) must be undertaken to assess other known causes of neonatal seizures. A detailed description of delivery will help in define hypoxic-ischemic encephalopathy-related seizures, requiring neuroprotection with beginning of neonatal hypothermia within 6 h from birth [5].

### Neonatal hyponatremia

Neonatal electrolyte disorders account for 10 % of seizures at birth; [2] mild hyponatremia, defined as a serum concentration of sodium of <133–135 mEq/L, occurs in 25 % of ill newborns, while moderate hyponatremia (sodium <130 mEq/L) occurs in 1 % [6].

Physiopathology of seizures secondary to hyponatremia recognizes an increase of cerebral water which, if acute (ie, acute hyponatremia) leads to cellular edema and subsequent encephalopathy, seizures, respiratory arrest, and coma. On the contrary, chronic hyponatremia is better tolerated because of the brain capability to produce the so-called idiogenic osmoles, with tend to normalize intracellular water content [7]. Chronic hyponatremia may in turn be associated to poor neonatal growth [8] and/or to sensorineural hearing loss.

Typically, newborns develop hyponatremia due to excessive renal sodium loss and/or kidney inability to excrete free water; in addition, hyponatremia may be sustained by high neonatal vasopressin levels [6].

Basing on plasma osmolarity, hyponatremia in newborns can be classified into hysotonic (280–295 mOsm/L; extremely rare in the newborn and generally due to laboratory errors, although possibly caused by rise of plasmatic lipids or proteins), hypertonic (>295 mOsm/L; occasionally found in newborns, particularly those with concomitant hyperglycemia), and hypotonic (<280 mOsm/L; the most frequent form in newborns, furtherly classified into three subcategories according to patient’s extracellular fluid volume status: hypovolemic, euvolemic and hypervolemic) [6].

According to time of onset, neonatal hyponatremia can be classified into acute (onset within 48 h) and chronic (developing after more of 48 h).

Acute hyponatremia, in our case, reflected maternal pre-delivery hyponatremia (probably induced by excessive hydration during labor). Neonatal serum electrolytes in the first hours of life reflect indeed those of mother, since microelements tend to balance across placental interface.

Other causes of neonatal hyponatremia include continuous glucagon infusion [9], accidental administration of oxytocin [10], congenital hypothyroidism [11], and congenital adrenal hyperplasia [12].

Causes of maternal hyponatremia include excessive oral water intake during labour [13, 14], iatrogenic maternal fluid overload [15, 16], maternal medications like oxytocin, diuretics, ACE inhibitors, antidepressants like selective serotonin reuptake inhibitors [SSRI’s], synthetic recreational drugs like ecstasy.

In our case, the first maternal blood sodium available (123 mEq/L) was obtained during labor and was already extremely low; unfortunately, maternal blood sodium value before labor commencement was not available. The woman was always asymptomatic; her blood sodium value rose to and remained stable at around 140 mmol/L after maternal sodium correction, which indirectly suggests the absence of maternal conditions predisposing to hyponatremia.

## Conclusions and final remarks

### What should the Neonatologist retain

Neonatal seizures are always an emergent issue in NICUs. First line diagnostics should include a blood gas analysis and a blood glucose test, with following appropriate correction if indicated; antiepileptic drugs should be started if seizures don’t cease despite metabolic derangements correction, and therapeutic hypothermia initiated if appropriate.

Further diagnostics should include cerebral ultrasound and/or magnetic resonance imaging, electroencephalogram, microbiology tests, laboratory ammonium and lactate to assess other known causes of neonatal seizures.

In the event of acute hyponatremia-related seizures, rapid sodium correction is mandatory since the risk of sequelae is greater than that of myelinolysis; according to most Authors, plasma sodium should be raised quickly until 125 mmol/L (or, anyhow, blood sodium target must be that which allows the patient to become asymptomatic) and then corrected slowly (not more than 8–10 mmol/L in the first 24 h –i.e., 0.3–0.4 mmol/L/h) to avoid both cerebral oedema and the risk of myelinolysis) [6], with at least two-hourly monitoring of sodium levels. Although little evidence-based information on the appropriate rate at which to correct severe hyponatremia in neonates exist, previously cited velocities of blood sodium correction (0.3–0.4 mmol/L/h) [6] seem consistent with guidelines about rapid correction of severe hyponatremia in older children, which suggest a maximum correction velocity for blood sodium of 0.5 mmol/L/h [17].

Fluid restriction should also be undertaken as a corrective measure of hyponatremia in the term newborn if history suggests low blood sodium secondary to maternal water intoxication; this is supported by the observation that these newborns manifest an excess weight loss after birth when compared to isonatriemic newborns [15]. On the contrary, fluid restriction is not to be considered as a first line treatment for hyponatremia of low birth weight [LBW] and very low birth weight [VLBW] preterm newborns: these patients in fact recognize different mechanisms leading to hyponatremia, such as increased sodium demand (particularly in the “catch up” growth phase) and increased sodium renal losses due to immature tubular function [18].

In order to prevent hyponatremia in these patients, IV sodium supplementation start should actually be considered when blood sodium becomes lower than 140 mmol/L, which usually occurs on day of life two or three; then, a minimum of 4 mmol/kg/die sodium supplementation is warranted to grant an adequate growth. Other possible sodium losses (eg, nasogastric tube, stomies, surgical drainages) also must be considered and replaced [18].

On the contrary, healthy term newborn do not routinely need sodium supplementation due to renal tubule maturation, which grants a much better hydroelectrolytic homeostasis for these patients. Newborn outcomes of symptomatic hyponatremia include convulsions, apnoea, cyanotic spells, respiratory distress, feeding difficulty, and excess weight loss after birth; [15] several reports anyway describe a generally good long-term neurologic outcome in these patients [13, 14, 19] – provided that hyponatremia is rapidly detected and treated as the underlying cause of neonatal seizures.

### What should the obstetric retain

Several randomized trials [20–23] have established that the routine administration of oral and/or IV fluids to keep women adequately hydrated during labour may reduce the period of contraction and relaxation of the uterine muscle, and may ultimately reduce the duration of the labour. Labor patients hydration protocols, anyway, are extremely variable from Centre to Centre. A recent systematic review [24] based on nine randomized trials with 1781 women does not provide robust evidence to recommend routine administration of intravenous fluids. Additionally, in trials where oral fluids were not restricted there was considerable variation in the amount of oral fluid consumed by women in different arms of the same trial, and between different trials [24].

What is clear is that, when a more than mild hyponatremia is detected in pregnant women at pre-delivery blood tests, a thorough maternal history should be taken about hydration status, sodium-active drugs use (diuretics, ACE inhibitors, antidepressants like SSRI’s, synthetic recreational drugs like ecstasy), sodium-affecting pathologies (heart failure, adrenal insufficiency, renal failure, hypothyroidism, cystic fibrosis).

Labor hydration itself (particularly if conducted with sodium-free solutions like 5 % or 10 % dextrose) can lead to acute hyponatremia in previously normosodiemic women; [15] consequently, even in women who have normal blood sodium at admittance, a basal sodium supplementation should be given along with IV or oral hydration during labor, in order to prevent iatrogenic maternal (and consequently, neonatal) hyponatremia.

Obstetric must also be aware that neonatal hyponatremia has also been reported with underwater births [25–27].

Feto-maternal equilibrium through placental homeostasis is a well-established concept, so Obstetrics must be aware that any maternal dyselectrolytemia will reflect itself in the newborn. Thus, efforts should be made to correct maternal electrolyte imbalance (here discussed, hyponatremia) before delivery, particularly if local protocols consider oral or IV fluids administration to shorten labor; if maternal electrolyte correction is not possible due to emergent delivery, it is anyway important to warn the neonatologist about the type and gravity of maternal metabolic derangement, so that appropriate monitoring and timely correction of dyselectrolytemia may be performed on the newborn after birth.

## Informed consent

Written informed consent was obtained from the patient’s parents for publication of this Case report and any accompanying images. A copy of the written consent is available for review by the Editor-in-Chief of this journal.
